# Strategies to counter disinformation for healthcare practitioners and policymakers

**DOI:** 10.1002/wmh3.487

**Published:** 2021-11-24

**Authors:** Julian H. Neylan, Sonny S. Patel, Timothy B. Erickson

**Affiliations:** ^1^ Harvard Humanitarian Initiative T.H. Chan Harvard School of Public Health Cambridge Massachusetts USA; ^2^ Department of Medicine and Health, Sydney School of Health Sciences The University of Sydney New South Wales Australia; ^3^ Division of Medical Toxicology, Department of Emergency Medicine Mass General Brigham, Harvard Medical School Boston Massachusetts USA

**Keywords:** disinformation, global health, health communication, health policy, misinformation

## Abstract

Medical disinformation has interfered with healthcare workers' ability to communicate with the general population in a wide variety of public health contexts globally. This has limited the effectiveness of evidence‐based medicine and healthcare capacity. Disinformation campaigns often try to integrate or co‐opt healthcare workers in their practices which hinders effective health communication. We describe a critical overview of issues health practitioners and communicators have experienced when dealing with medical disinformation online and offline as well as best practices to overcome these issues when disseminating health information. This article lists disinformation techniques that have yet to be used against the medical community but need to be considered in future communication planning as they may be highly effective. We also present broad policy recommendations and considerations designed to mitigate the effectiveness of medical disinformation campaigns.

## Highlights

- Medical practitioners must be aware they can be co‐opted by disinformation campaigns.
- The medical community needs to be prepared for emerging technologies that will allow for sophisticated disinformation campaigns.
- Adequate cyber security infrastructure is essential to combatting disinformation

## Resumen

La desinformación médica ha interferido con la capacidad de los trabajadores de la salud para comunicarse con la población en general en una amplia variedad de contextos de salud pública a nivel mundial. Esto ha limitado la eficacia de la medicina basada en la evidencia y la capacidad sanitaria. Las campañas de desinformación a menudo intentan integrar o cooptar a los trabajadores de la salud en sus prácticas, lo que dificulta la comunicación efectiva de la salud. Describimos una descripción general crítica de los problemas que los profesionales de la salud y los comunicadores han experimentado al lidiar con la desinformación médica en línea y fuera de línea, así como las mejores prácticas para superar estos problemas al difundir información de salud. Este artículo enumera las técnicas de desinformación que aún no se han utilizado contra la comunidad médica, pero que deben tenerse en cuenta en la planificación de comunicaciones futuras, ya que pueden ser muy efectivas. También presentamos amplias recomendaciones y consideraciones de políticas diseñadas para mitigar la efectividad de las campañas de desinformación médica.

## 摘要

医疗虚假信息已在全球广泛的公共卫生情境下干扰了医疗工作者对大众进行医疗传播的能力。这限制了基于证据的药物的有效性以及医疗能力。虚假信息活动经常试图在其实践中融入或拉拢医疗工作者，进而阻碍有效的卫生传播。我们批判概述了卫生从业人员和传播者在线上和线下应对医疗虚假信息时所经历的一系列问题，以及在传播卫生信息时用于克服这些问题的最佳实践。本文列出了一系列还未用于医疗界、但需要在未来传播规划时加以考量的虚假信息技术，因为这些技术可能具有高效性。我们还提出了广泛的政策建议和考量，用于缓解医疗虚假信息活动的有效性。

## INTRODUCTION

Over the past year, medical misinformation and disinformation have been on the rise in countries across the globe aimed at various targeted audiences. This has countered the ability of healthcare practitioners to disseminate accurate information to their patients and has limited the effectiveness of evidence‐based medicine. Misinformation is defined “information that is contrary to the epistemic consensus of the scientific community regarding a phenomenon” while disinformation is an intentional effort to spread misinformation (Swire‐Thompson & Lazer, 2020). Mainly, the perception of the public has been broadly targeted, but in notable instances, the healthcare infrastructure was disrupted in several specific countries such as Ukraine, Brazil, and the United States (Brown, 2020; Patel et al., 2020; Ricard & Medeiros, 2020).

The medical community has been making strides to reduce incorrect or misleading information. However, successful disinformation campaigns from both state actors and from actors in civil society have shaken the confidence of the public in healthcare systems. Many members of the medical community have actively promoted the idea that healthcare workers should establish online presences to counter disinformation by incorporating digital health literacy programs (Rubin, 2019). At present, digital health and media literacy are becoming more accessible; however, it has been exceedingly difficult to gauge the efficacy of health literacy programs to address the complex nature of misinformation (Jackson et al., 2021; Swire‐Thompson & Lazer, 2020). Health care workers need to be aware of the threats disinformation campaigns can pose against them when they establish an online presence or attempt to counter disinformation on behalf of their patients. Furthermore, disinformation has varied effects across varied contexts due to cultural, sociological, and technological differences. For example, areas with lower trust in media are more susceptible to belief in disinformation (Bontcheva et al., 2020). With this in mind, our best practices and policy recommendations are not for any particular context but represent broad steps that public health officials, governments, and organizations can take against disinformation. Much of the study of disinformation is communicated in English with a focus on western contexts. Therefore, more research and intervention are necessary to establish what is effective in other contexts outside the United States and globally.

In this commentary, we describe a critical overview of medical information issues health practitioners commonly encounter when dealing with disinformation online and offline, as well as best practices to overcome these challenges. We also identify disinformation tactics and technologies which can be utilized against the medical community. Finally, we present longer‐term policy recommendations designed to make these disinformation tactics less effective in the future.

### Existing practices in spreading medical disinformation

A large portion of disinformation surrounding health information does not involve members of the medical community. Social media and the vast majority of the internet consists of user‐generated content with different sites having varying degrees of moderation. Many users post unverified medical information, whether with malicious intent or because they are misguided. When medical practitioners attempt to disseminate public health information, they should be aware that they may be co‐opted by disinformation campaigns. Additionally, disinformation campaigns often originate from healthcare providers within the medical community, and public health communicators need to be able to respond to these qualified experts who intentionally spread discredited information.

### Co‐opting medical practitioners

Medical practitioners and researchers often attempt to combat disinformation but instead become unknowing actors in disinformation campaigns themselves. For example, a well‐meaning physician took a photo of himself in front of empty hospital beds which people online misinterpreted as the COVID‐19 pandemic being a “hoax”, when in fact, he was in front of additional bedding which was being set up due to increased patient volume and hospital overcrowding from COVID‐19 disease (Dupuy, 2020). There are innumerable other examples among conspiracists which feature videos or pictures of members of the medical community which are labeled with incorrect contexts to promote false narratives. Additionally, pictures can be altered, sound bites taken out of context, and videos selectively edited to give false impressions.

Conspiracists frequently view public health messaging as part of sinister conspiracies to manipulate the population. As such, the credentials of medical professionals are often seen as evidence that they are complicit in these conspiracies. Conspiracists will actively co‐opt public health messaging to counter official narratives. In March 2020, a physician created #DoctorsSpeakUp to speak out against the anti‐vax community, instead the anti‐vax community hijacked the hashtag and used it to ask doctors to speak out in favor of their conspiracies. The hashtag then “went viral” and began widely trending after it was co‐opted (Morris, 2020).

Even if practitioners are not directly co‐opted, controversial findings can be misinterpreted or emphasized to generate fear. In the Kremlin‐backed influence campaigns against the COVID‐19 Pfizer vaccine, they emphasized known vaccine side effects mixed with complete fabrications to dissuade the public from receiving the vaccine while encouraging them to wait for the “superior” Russian‐sponsored Sputnik V vaccine to become available (Volz & Dustin, 2021).

Practitioners need to be cautious with what information they enter online as their content can be readily altered or misconstrued. On the internet, nuanced debates and discussions are few and far between and practitioners should assume nothing regarding the reliability, intent, or comprehension level of their audience. There should be no expectations that others online will be reasonable, logical, or sympathetic. Additionally, online campaigns can utilize tools such as automated fake accounts to drown out health policy content they disagree with. A study by researchers at Carnegie Melon University in May 2020 found that nearly half of all Twitter accounts promoting reopening campaigns after COVID‐19 lockdowns in the United States were likely internet bots (Alvino Young, 2020). When practitioners see that their content is being misused, they should inform social media platforms and other relevant authorities as quickly as possible to avoid further dissemination. In the longer term, there should be health policy aimed at increasing scientific literacy so that people are able to better understand the argumentation and reasoning of medical practitioners and identify logical fallacies in conspiratorial thinking.

### Pseudoscience promoted by medical professionals

There are some medical professionals who choose to misuse their titles so that they can lend credibility to unscientific or outright false claims. A former virologist was featured on the conspiracy documentary “*Plandemic*” which was viewed tens of millions of times globally and asserted that COVID‐19 disease could be prevented by exposure to sunlight. In late 2020, the former vice president of Pfizer co‐authored a petition that, without evidence, concluded that COVID‐19 vaccines could cause infertility in women (Stecklow & Macaskill, 2021). The Center for Countering Digital Hate found that up to 65% of anti‐vaccine content was spread by only a dozen people, of which, three were physicians (The Center for Countering Digital Hate, 2021). Medicine has a long history of people misusing their credentials to promote unscientific views. With the expansion of social media, this will continue to be a perennial problem beyond the current pandemic.

Physicians must be prepared to speak out about those in the medical profession who spread conspiracies. Most major social media platforms have reporting features which allow individuals to flag mis or disinformation. Social media platforms can screen for medical disinformation and have been working with organizations like the World Health Organization (WHO) and other public health authorities (Meyer & Alaphilippe, 2021). However, more medical organizations and informed healthcare professionals are needed to assist these efforts as social media platforms are ill‐equipped to counter individuals spreading disinformation who have a reasonable claim to expertise. For this reason, unbiased, creditable medical professionals should work with social media companies to properly verify information.

### Potential disinformation threats

The nature of online disinformation is that it is ever changing and rapidly adapting to countermeasures. The medical community including public health communicators, medical practitioners, and administrators need to be prepared for existing trends in disinformation as well as for how emerging technologies will impact health communication. Below are a few information gaps currently not addressed in health communication strategies that demonstrate current vulnerabilities. In health policy terms, there needs to be concrete communication strategies in place to prepare for and counter new developments in the promotion of disinformation. Additionally, beyond being aware of these threats, healthcare practitioners, social media platforms, civil society, and government organizations need to collaborate and develop tools for a resilient information ecosystem that can combat ever adapting technological threats without restricting public free speech (Felten & Nelson, 2019). Tools such as artificial intelligence (AI) detection of altered media with improved cyber security of the healthcare infrastructure can directly counter the disinformation techniques addressed below.

#### Altered websites

Legitimate sources can be replicated and manipulated online. For example, in 2017 a disinformation campaign managed to copy the web design of an influential Harvard University Center and several news outlets including Le Soir and The Guardian and they inserted their own false articles which were designed to discredit US and Saudi Arabian policies in the Middle East (Lim et al., 2019). Actors spreading disinformation can do this by copying the source code of websites to make them appear exactly as they would normally, copying the same design, advertisements and putting in functional “share” buttons to social media. The URLs can be made to look similar to the websites they are imitating with subtle differences in typos, letters that look similar (e.g., using the lowercase of the letter L “l” instead of the uppercase of the letter “I”), and the top‐level domain (e.g., from.info to.net). These fake web pages may even redirect to the actual source they are trying to imitate so that clicking on sections of the page will lead you to the actual site. Prominent high‐impact medical sources like peer‐reviewed journals need to be aware that they can be imitated by factions who are willing to use their platform to spread medical inaccuracies. This goes beyond “predatory” journals to potentially any online medical source the public views as legitimate. As a result, healthcare providers must closely check URLs to see if they are correct. Cross‐checking information with multiple sources is important to discern if the information is genuine. Whenever one of these false web pages is identified, the organization being imitated needs to quickly respond to any online platforms where the links to the false page are disseminated so that they can remove the inaccurate content.

#### Deep fakes

“Deep fakes” or artificial intelligence‐generated false videos came into prominence in 2017 and have become increasing accessible for nonvisual effects specialists to use. While extremely convincing, deep fake videos still require professional visual effects (VFX) designers and actors, although there have been cases where amateurs have created deep fakes to damage reputations of others. Rogue companies are working to develop deep fake technology that can be used in real‐time. Thus far, deep fakes have not been used to target the medical community, but health practitioners need to be prepared for this eventuality. Most social media platforms have some form of ban on artificial intelligence generated false videos and images, but they require tools to properly identify deep fakes. Experts are creating tools which will allow artificial intelligence (AI) to automatically detect when deep fake technology is used; currently this technology is still in development.

#### Leaked or altered information after a cyber security breach

The medical field has been highly impacted by cyber‐attacks. A 2017 survey of the American Medical Association (AMA) found that 83% of physicians claimed their practices had experienced some form of cyber‐attack (AMA Staff News Writer, 2021). Sensitive healthcare information is frequently revealed in hacks. One list was publicly posted on 4chan, an image‐based bulletin board where anyone can post comments and share images anonymously. This particular list was compiled from a series of hacks which had thousands of emails and passwords from the National Institute of Health (NIH), Center for Disease Control and Prevention (CDC), World Bank, and World Health Organization (WHO) (Wakefield, 2020). Often these attacks come in the form of “phishing”, where emails will impersonate legitimate sources to try to get access to information. In 2020, a hack for hire campaign targeting healthcare companies and consulting services used Gmail accounts impersonating the WHO to direct users to WHO lookalike websites where they were urged to sign up for alerts which required them to give personal information (Vavra, 2020). Disinformation campaigns have used information gained during cyber breaches and leaked altered versions of official documents to create false narratives. In one instance, journalist David Satter had his e‐mail hacked by the pro‐Russian hacktivist group Cyberberkut who proceeded to alter his documents and leak them online (Hulcoop et al., 2017). Additionally, information gained during leaks can be misinterpreted or represent incomplete scientific findings which if made publicly available, constitute a form of disinformation.

To combat this, effective cybersecurity requires ensuring that both hardware and software are well maintained and regularly updated. Medical personnel need to be informed about threats from malware, hackers, and viruses and need to be transparent when breaches do occur. Additionally, they should be educated regarding online impersonation and methods to discern genuine websites. Health documents should be backed‐up such that if leaked and altered, practitioners have original unaltered documents to refute the leak. When there is a breach, the healthcare professional should notify the related services targeted (e.g., Google, Facebook) as well as the local authorities as quickly as possible (See Table 1).

**Table 1:** Ten tips for healthcare practitioners about disinformation

1. Practitioners need to be cautious posting online ‐ anything said can be altered	6. Effective cybersecurity requires hardware and software be maintained and regularly updated ‐ out of date security software is more susceptible to online threats
2. Social media organizations need to be informed when videos or images are being used to promote disinformation	7. When a breach is detected contact relevant services as well as the local authorities
3. Be prepared to speak out about those in the medical profession who spread conspiracies.	8. Backup documents so that if they are leaked or altered the original documents can refute the disinformation
4. Closely check URLs to see if they are legitimate	9. Medical staff personnel need to be informed about the threats from malware, hackers, viruses, and those phishing for sensitive information
5. Cross check information with multiple sources to better discern if information is genuine	10. Conspiracies are often best countered through compassion and empathy rather than fact or argumentation.

### How to deal with the nontechnical side of disinformation

Most of the factors that can mitigate disinformation go beyond the field of medicine or technology but with public health communication. At the root of disinformation is often miscommunication and distrust, which require more than just several correct facts to resolve.

Health practitioners should be made aware of the common disinformation which is easily accessible to patients. However, given the sheer volume of disinformation, this will be a difficult undertaking. Health communicators must work to inform practitioners about the most common or dangerous narratives that their patients have been exposed to through means of periodically published condensed reviews of disinformation. Additionally, practitioners must be aware that the cold facts are not enough. Fact checking has been shown to have positive effects in terms of correcting inaccurate information, however, it is far less effective at altering beliefs and actions (Barrera et al., 2020). Many scholars blame this on “motivated reasoning” where audiences ignore information that does not fit their preconceptions (Bardon, 2019). Other scholars have suggested that a lack of reasoning is to blame for people's inability to differentiate between what is true and what is false (Pennycook & Rand, 2019). Furthermore, factual corrections can lead to a “backfire effect” where people reinforce their incorrect views in light of contradictory information (Nyhan & Reifler, 2010). Studies claiming to identifying this effect or phenomenon have been recently disputed (Guess & Coppock, 2020). There are effective means to reach patients who believe in disinformation campaigns. Prompting analytical consideration of conspiracies to examine inconsistencies can lead to “the elusive backfire effect” where people cease to believe conspiratorial thinking (Wood & Porter, 2019).

Academic and healthcare training workshops address how to deal with patients who display conspiratorial thinking, emphasizing that it is best to always show consistent messaging, empathy, and understanding rather than argumentation (Abbasi, 2021). Healthcare policymakers need to incorporate training and educational materials for healthcare providers to better communicate with their patients when exposed to disinformation.

#### Policy recommendations to combat health disinformation

Public Health disinformation constitutes a significant challenge for policymakers as actions taken against disinformation can infringe upon freedom of speech. Educational programs that encourage critical thinking and allow users to identify disinformation for themselves are an attractive solution as no outside bodies need to remove or add disclaimers to content. There have been large increases in the accessibility of digital health however, there are barriers and disparities to digital literacy, such as “inequitable access to digital technologies; and low general and domain‐specific literacies.” (Jackson et al., 2021). Additionally, it is exceedingly difficult to gauge the efficacy of these health literacy programs (Swire‐Thompson & Lazer, 2020). Additional research is necessary to find the most effective solutions to the evolving problem of disinformation. Policymakers should support research on disinformation to have multi‐stakeholder collaborations between vested civil society, health care, and technology actors. Disinformation is a global problem and policymakers must coordinate transnational digital health literacy programs concerning disinformation threats, including those that come from newly generated technologies. We recommend increased international collaboration between stakeholders affected by disinformation to improve learnings and best practices (Felten & Nelson, 2019). Additionally, policymakers need to consider that government‐led initiatives to counter disinformation may be impartial as they have proven to be most effective only when they are transparent and not unidirectional strategic communications serving self‐motivated political interests (Bontcheva et al., 2020).

Besides these measures, it will be essential in the long term for policymakers to address the structural factors which facilitate disinformation online. Currently, information from unofficial sources is frequently conflated with those from official sources. It is crucial for social media platforms to identify and act against content harmful to public health initiatives and implement features that allow for easier distinction between official and unofficial content (such as banners or notifications that identify sources) (Simpson & Conner, 2020). However, it is equally important that platforms exercise caution when implementing actions that prioritize only official sources as their sites should also be able to serve scientists who want to collaborate and share findings that may have yet to be officially verified. Policy makers can push for social media platforms to emphasize and amplify content from public health sources. Additionally, credible sources should inform the content moderation efforts of social media platforms. Policymakers can work to ensure that platforms work collaboratively with public health authorities to have the most current information for fact‐checkers. These broad policy recommendations and considerations are ultimately designed to mitigate the effectiveness of medical disinformation campaigns.

## ETHICS STATEMENT

This article is an original work, which has not been published before, and is not being considered for publication elsewhere in its final form, in either printed or electronic media. Any republication of the content will not constitute redundant publication, will not breach copyright, and will reference the original publication.

## References

[wmh3487-bib-0001] Abbasi, J. (2021). COVID‐19 conspiracies and beyond: How physicians can deal with patients' misinformation. Journal of the American Medical Association, 325(3), 208–210. 10.1001/jama.2020.22018 33377900

[wmh3487-bib-0002] Alvino Young, V. (2020). Nearly half of the Twitter accounts discussing ‘Reopening America’ may be bots. Carnegie Mellon University. Retrieved 15 July 2021 from https://www.cmu.edu/news/stories/archives/2020/may/twitter-bot-campaign.html

[wmh3487-bib-0003] AMA Staff News Writer . (2021). 8 in 10 doctors have experienced a cyberattack in practice. American Medical Association. Retrieved 15 July 2021 from https://www.ama-assn.org/practice-management/sustainability/8-10-doctors-have-experienced-cyberattack-practice

[wmh3487-bib-0004] Bardon, A. (2019). The truth about Denial: Bias and self‐deception in science, politics, and religion. Oxford University Press. 10.1093/oso/9780190062262.001.0001

[wmh3487-bib-0005] Barrera, O. , Guriev, S. , Henry, E. , & Zhuravskaya, E. (2020). Facts, alternative facts, and fact checking in times of post‐truth politics. Journal of Public Economics, 182, 104123. 10.1016/j.jpubeco.2019.104123

[wmh3487-bib-0006] Bontcheva, K. , Posetti, J. , Teyssou, D. , Meyer, T. , Gregory, S. , Hanot, C. , & Maynard, D. (2020). Balancing act: Countering digital disinformation while respecting freedom of expression broadband commission research report on ‘freedom of expression and addressing disinformation on the Internet’. ITU, UNESCO & Broadband Commission for Sustainable Development. Retrieved 15 July 2021 from https://en.unesco.org/publications/balanceact

[wmh3487-bib-0007] Brown, A. M. (2020). Digital disinformation is a threat to public health. Lerner Center for Public Health Promotion: Population Health Research Brief Series, 31 ​. https://surface.syr.edu/lerner/31

[wmh3487-bib-0008] Dupuy, B. (2020). Reno doctor's selfie hijacked to imply COVID is a hoax. *The Mercury News*. Retrieved 15 July 2021 from https://www.mercurynews.com/2020/12/02/reno-doctors-selfie-hijacked-to-imply-covid-is-a-hoax

[wmh3487-bib-0009] Felten, C. , & Nelson, A. (2019). Countering misinformation with lessons from public health. Center for Strategic & International Studies. Retrieved 15 July 2021 from https://www.csis.org/countering-misinformation-lessons-public-health

[wmh3487-bib-0010] Guess, A. , & Coppock, A. (2020). Does counter‐attitudinal information cause backlash? Results from three large survey experiments. British Journal of Political Science, 50(4), 1497–1515. 10.1017/S0007123418000327

[wmh3487-bib-0011] Hulcoop, A. , Scott‐Railton, J. , Tanchak, P. , Brooks, M. , & Deibert, R. (2017). Tainted leaks: Disinformation and phishing with a Russian Nexus. The Munk School of Global Affairs & Public Policy, The University of Toronto. Retrieved 15 July 2021 from https://citizenlab.ca/2017/05/tainted-leaks-disinformation-phish/

[wmh3487-bib-0012] Jackson, D. N. , Trivedi, N. , & Baur, C. (2021). Re‐prioritizing digital health and health literacy in healthy people 2030 to affect health equity. Health Communication, 36(10), 1155–1162. 10.1080/10410236.2020.1748828 32354233

[wmh3487-bib-0013] Lim, G. , Maynier, E. , Scott‐Railton, J. , Fittarelli, A. , Moran, N. , & Deibert, R. (2019, 2019/05/14). Burned after reading: Endless mayfly's ephemeral disinformation campaign. Retrieved 15 July 2021 from. https://citizenlab.ca/2019/05/burned-after-reading-endless-mayflys-ephemeral-disinformation-campaign/

[wmh3487-bib-0014] Meyer, T. , & Alaphilippe, A. (2021). One year onward: Platform responses to COVID‐19 and US Elections Disinformation in Review. EU Disinfo Lab. Retrieved 15 July 2021 from https://www.disinfo.eu/publications/one-year-onward-platform-responses-to-covid-19-and-us-elections-disinformation-in-review

[wmh3487-bib-0015] Morris, S. (2020). #DoctorsSpeakUp trends on Twitter as anti‐vaccine movement hijacks hashtag intended to combat misinformation. *Newsweek*. Retrieved 15 July 2021 from https://www.newsweek.com/doctors-speak-twitter-trend-anti-vaxxers-vaccine-hashtag-1490692

[wmh3487-bib-0016] Nyhan, B. , & Reifler, J. (2010). When corrections fail: The persistence of political misperceptions. Political Behavior, 32(2), 303–330. 10.1007/s11109-010-9112-2

[wmh3487-bib-0017] Patel, S. , Moncayo, O. , Conroy, K. , Jordan, D. , & Erickson, T. (2020). The landscape of disinformation on health crisis communication during the COVID‐19 pandemic in Ukraine: Hybrid warfare tactics, fake media news and review of evidence. Journal of Science Communication, 19(5), A02. 10.22323/2.19050202 PMC8425291 34504624

[wmh3487-bib-0018] Pennycook, G. , & Rand, D. G. (2019). Lazy, not biased: Susceptibility to partisan fake news is better explained by lack of reasoning than by motivated reasoning. Cognition, 188, 39–50. 10.1016/j.cognition.2018.06.011 29935897

[wmh3487-bib-0019] Ricard, J. , & Medeiros, J. (2020). Using misinformation as a political weapon: COVID‐19 and Bolsonaro in Brazil. Harvard Kennedy School Misinformation Review, 1(3)​, 10.37016/mr-2020-013 PMC12424044 40948887

[wmh3487-bib-0020] Rubin, R. (2019). Getting social: Physicians can counteract misinformation with an online presence. Journal of the American Medical Association, 322(7), 598–600. 10.1001/jama.2019.10779 31365030

[wmh3487-bib-0021] Simpson, E. , & Conner, A. (2020). Fighting coronavirus misinformation and disinformation preventive product recommendations for social media platforms. Center for American Progress. Retrieved 15 July 2021 from https://www.americanprogress.org/issues/technology-policy/reports/2020/08/18/488714/fighting-coronavirus-misinformation-disinformation/

[wmh3487-bib-0022] Stecklow, S. , & Macaskill, A. (2021). The ex‐Pfizer scientist who became an anti‐vax hero. *Reuters*. Retrieved 15 July 2021 from https://www.reuters.com/investigates/special-report/health-coronavirus-vaccines-skeptic/

[wmh3487-bib-0023] Swire‐Thompson, B. , & Lazer, D. (2020). Public health and online misinformation: Challenges and recommendations. Annual Review of Public Health, 41, 433–451. 10.1146/annurev-publhealth-040119-094127 31874069

[wmh3487-bib-0024] The Center for Countering Digital Hate . (2021). The disinformation dozen. Retrieved 15 July 2021 from https://www.counterhate.com/disinformationdozen

[wmh3487-bib-0025] Vavra, S. (2020). Google finds hack‐for‐hire firms posing as World Health Organization via email. *Cyberscoop*. Retrieved 15 July 2021 from https://www.cyberscoop.com/coronavirus-phishing-scheme-google-india-world-health-organization/

[wmh3487-bib-0026] Volz, M. R. G. , & Dustin, V. (2021). WSJ news exclusive: Russian disinformation campaign aims to undermine confidence in Pfizer, other Covid‐19 vaccines, U.S. Officials Say. *Wall Street Journal*. Retrieved 15 July 2021 from https://www.wsj.com/articles/russian-disinformation-campaign-aims-to-undermine-confidence-in-pfizer-other-covid-19-vaccines-u-s-officials-say-11615129200

[wmh3487-bib-0027] Wakefield, J. (2020). Coronavirus: Health leaders' credentials dumped online. *BBC News*. Retrieved 15 July 2021 from https://www.bbc.com/news/technology-52381356

[wmh3487-bib-0028] Wood, T. , & Porter, E. (2019). The elusive backfire effect: Mass attitudes' steadfast factual adherence. Political Behavior, 41(1), 135–163. 10.1007/s11109-018-9443-y

